# Differential effects of selective and non-selective cyclooxygenase inhibitors on fecal microbiota in adult horses

**DOI:** 10.1371/journal.pone.0202527

**Published:** 2018-08-23

**Authors:** Canaan M. Whitfield-Cargile, Ana M. Chamoun-Emanuelli, Noah D. Cohen, Lauren M. Richardson, Nadim J. Ajami, Hannah J. Dockery

**Affiliations:** 1 Department of Large Animal Clinical Sciences, College of Veterinary Medicine & Biomedical Sciences, Texas A&M University, College Station, Texas, United States of America; 2 The Alkek Center for Metagenomics and Microbiome Research, Baylor College of Medicine, Houston, Texas, United States of America; University of Calgary, CANADA

## Abstract

Non-steroidal anti-inflammatory drugs (NSAIDs) are routinely used in both veterinary and human medicine. Gastrointestinal injury is a frequent adverse event associated with NSAID use and evidence suggests that NSAIDs induce gastrointestinal microbial imbalance (*i*.*e*., dysbiosis) in both animals and people. It is unknown, however, whether cyclooxygenase (COX)-2-selective NSAIDs induce dysbiosis, or if this phenomenon occurs in horses administered any class of NSAIDs. Therefore, our objectives were to determine whether the composition and diversity of the fecal microbiota of adult horses were altered by NSAID use, and whether these effects differed between non–selective and COX-2-selective NSAIDs. Twenty-five adult horses were randomly assigned to 1 of 3 groups: control (n = 5); phenylbutazone (n = 10); or, firocoxib (n = 10). Treatments were administered for 10 days. Fecal samples were collected every 5 days for 25 days. DNA was extracted from feces and the 16S rRNA gene amplified and sequenced to determine the composition of the microbiota and the inferred metagenome. While the fecal microbiota profile of the control group remained stable over time, the phenylbutazone and firocoxib groups had decreased diversity, and alteration of their microbiota profiles was most pronounced at day 10. Similarly, there were clear alterations of the inferred metagenome at day 10 compared to all other days, indicating that use of both non-selective and selective COX inhibitors resulted in temporary alterations of the fecal microbiota and inferred metagenome. Dysbiosis associated with NSAID administration is clinically relevant because dysbiosis has been associated with several important diseases of horses including abdominal pain (colic), colitis, enteric infections, and laminitis.

## Introduction

Non-steroidal anti-inflammatory drugs **(NSAIDs)** are one of the most frequently consumed pharmaceuticals in the world [1]. In veterinary medicine, NSAIDs are second only to parasiticides in frequency of use [2]. In equine veterinary medicine, the exact prevalence of NSAID use is unknown; however, clinical experience indicates that NSAIDs are used ubiquitously in horses for their anti-inflammatory and analgesic properties. Although NSAIDs are used routinely in both human and veterinary medicine, their use has been associated with numerous adverse effects including NSAID-induced gastrointestinal (**GI**) injury. In the GI tract, NSAID-induced injury results in 2 clinical syndromes: 1) NSAID-induced gastropathy, affecting the stomach and proximal duodenum; and, 2) NSAID enteropathy, affecting the lower GI tract *distal to the duodenum* [3, 4].

Although detection and management strategies for NSAID-induced gastropathy are well documented, effective diagnosis and treatment strategies for NSAID enteropathy are lacking in both people and animals [3, 4]. In the United States alone, NSAID enteropathy results in approximately 100,000 hospitalizations and 16,500 deaths each year [5]. An additional 2/3 of both short- and long-term NSAID users develop subclinical or undiagnosed distal small intestinal lesions [6]. Although epidemiological data are lacking, NSAID enteropathy occurs in horses primarily as right dorsal colitis, which, similar to NSAID enteropathy in people, is difficult to diagnose and manage [7, 8].

In horses, as in people, changes in the GI microbiota are linked to an array of both intestinal and non-intestinal diseases [9–12]. Interestingly, therapeutic doses of NSAIDs can result in a dysbiosis that drastically alters the GI microbiota of people and laboratory animals [8, 13–15]. It has been consistently demonstrated that the primary bacterial phyla in mammalian GI tracts (including horses) are *Bacteroidetes* and *Firmicutes* [16]. Making comparisons between human and horse microbiota is difficult because of the relative paucity of studies examining the equine microbiota and because of differences in techniques, such that the specific ratios of major bacterial phyla vary among studies and health conditions of subjects. NSAID-induced dysbiosis is characterized most often by a reduction of the predominately Gram-positive phylum *Firmicutes* and a corresponding increase of Gram-negative bacteria, although this shift is not universal [8, 13–15, 17]. Several classes of *Firmicutes* that are decreased following NSAID therapy belong to a group of commensal *Clostridia* that are critically important in gut homeostasis, specifically members of *Clostridium* cluster *XIVa* and *Clostridium* cluster *IV* [15, 18]. The horse is a hind-gut fermenter and, as such, the GI microbiota is essential for digestion of nutrients and as an energy source [19, 20]. Therefore, any perturbation of the GI microbiota may have profound implications for the health and well-being of affected horses [9–12]. Given the implications of GI dysbioses in horses, the known effects of NSAIDs on the microbiota of people and other animals, and the highly prevalent use of NSAIDs in horses, it is possible that NSAID-induced dysbiosis might predispose horses to a number of diseases.

The analgesic and anti-inflammatory properties of NSAIDs are attributed to their inhibition of cyclooxygenase(s) (COX) enzymes. Inhibition of COX-1, the constitutively expressed isoform of the COX enzyme, is thought to contribute to damage to the GI tract by NSAIDs. Therefore, great effort has been made to generate NSAIDs that selectively inhibit COX-2, the inducible isoform considered to cause inflammation [21, 22]. Although the use of COX-2 selective NSAIDs in people has decreased the incidence of upper GI tract lesions, this approach has been less effective at reducing the incidence of NSAID enteropathy [23]. While the mechanisms for this lack of efficacy are unknown, one possible explanation is the increasingly recognized constitutive expression of COX-2 in the GI tract [24]. In rodent models, there is some evidence that COX-2 plays a role in gut mucosal homeostasis that is as, or more, important than COX-1, suggesting that inhibition of COX-2 is not necessarily a safer approach for the GI tract [22]. The differential effects of COX-selective vs. non-selective NSAIDs in horses remains unknown. Therefore, our objectives were to determine if: 1) the composition and diversity of the fecal microbiota (as a marker of distal GI microbiota) of adult horses are altered by NSAID use; and, 2) whether there are differential effects of COX-2-selective NSAIDs and non-COX-selective NSAIDs on the fecal microbiota of horses.

## Materials and methods

### Animals

The protocol for this study was approved by the University Institutional Animal Care and Use Committee (IACUC 2015–0382). Twenty-five healthy adult horses from the university teaching herd were utilized for this study. Inclusion criteria included age (5–20 years), breed (American Quarter Horse), and health status (free of signs of illness based on physical examination). Prior to enrollment in the study, all horses underwent a complete physical examination and medical history was reviewed. Horses were excluded if they failed to meet inclusion criteria, if physical examination revealed any evidence of illness, or if review of their medical record revealed a history of any illness or medications administered in the 6 months preceding enrollment in this study. To assign treatment groups, 5 horses were randomly selected from all horses that met inclusion criteria. These 5 horses were matched at a ratio of 4:1 with other horses based on age (+/- 2 years), sex, and weight (+/- 100 pounds) for a total of 25 horses. One horse from each quintet was randomly assigned to the control group, and the remaining horses from the quintet were randomly assigned in blocked manner to either firocoxib (n = 2 per quintet) or phenylbutazone (n = 2 per quintet). For 10 days prior to the start of the study, each horse was fed the same diet including hay from the same cutting. Fecal samples were collected by rectal palpation (using an individual rectal sleeve for each horse) every 5 days for 25 days beginning on day 0. Samples were collected in sterile containers and frozen at -80°C immediately after collection. NSAID administration began on day 1 and continued for 10 days. Based on the assigned group, horses were administered phenylbutazone (ButaPaste, Henry Schein, Dublin, OH; 4.4 mg/kg, orally once daily), firocoxib (Equioxx, Merial, Duluth, GA; 0.1 mg/kg, orally once daily), or vehicle (control). These dosages were selected on the basis of standard clinical practice for management of inflammatory conditions in horses (*e*.*g*., osteoarthritis) and in accordance with label directions [25–27]. We chose firocoxib as the representative COX-2 selective NSAID for use in this study because it is the only FDA-approved COX-2-selective NSAID available in the United States for use in horses. In addition, firocoxib is highly COX-2-selective with a COX-1:COX-2 ratio of 200, whereas phenylbutazone has a COX-1:COX-2 ratio of 1, indicating no COX selectivity [28, 29].

### Sequencing and analysis

DNA extraction, 16S rRNA gene PCR, and DNA sequencing were performed as previously described [30]. Briefly, 200 mg of feces were harvested from each frozen sample. Genomic DNA was extracted from the fecal samples using a commercially available fecal DNA extraction kit (QIAamp^®^ Fast DNA Stool Mini Kit, Qiagen, Germantown, MD) according to the manufacturer’s protocol with slight modifications. The frozen fecal aliquot was placed in a 2-ml tube that contained 1 ml Inhibitex^®^ buffer and 50 mg each of sterile/DNAase-free, 0.1- and 0.5-mm silica zirconium beads. Feces were homogenized for 135 seconds at 6.5 m/second with FastPrep^®^ FP120 cell disrupter (Qbiogene, Carlsbad, CA). The sample was then heated to 95°C for 5 minutes prior to following the manufacturer’s protocol for DNA extraction. DNA was suspended in tris-EDTA buffer (Integrated DNA Technologies, Coralville, IA) and stored at -80°C.

Amplification and sequencing of the V4 variable region of the 16S rRNA gene was performed at Baylor College of Medicine. Briefly, samples were barcoded and subjected to PCR amplification using primers 515F/806R directed towards the V4 hypervariable region of the 16S rRNA gene. Barcoded amplicon libraries were prepared and sequenced on a MiSeq (Illumina) following the manufacturer’s guidelines. The software Quantitative Insights Into Microbial Ecology (QIIME v1.9 (http://qiime.sourceforge.net) was used for data processing and analysis [31]. The raw sequence data were de-multiplexed, and low quality reads were filtered using database’s default parameters. Chimeric sequences were detected using Uchime and removed prior to further analysis [32]. Sequences were then assigned to operational taxonomic units (OTUs) using an open-reference OTU picking protocol in QIIME against the Greengenes database filtered at 97% identity [33–35]. To adjust for uneven sequencing depth among the samples, each sample was rarefied to an even sequencing depth prior to further analysis. Alpha rarefaction, beta diversity measures, richness, taxonomic summaries, and tests for significance were calculated and plotted using QIIME. Diversity indices were analyzed with generalized linear models using S-PLUS statistical software (Version 8.2, TIBCO, Inc., Seattle, WA). The dependent (outcome) variables were either the number of OTUs or phylogenetic diversity (PD) whole tree index; independent variables of time, group, and their interaction were modeled as fixed categorical variables (ordered in the case of time) and individual horse was modeled as a random effect. Post hoc multiple comparisons among groups and times were adjusted using the method of Sidak [36]. Model fit was assessed by visual inspection of plots of standardized residuals versus fitted values. Significance was set at P < 0.05. Principal coordinate analysis (PCoA) plots and UniFrac networks were performed with Phyloseq, an R-based package for analysis of microbiota sequencing data [37]. Differences in microbial communities within each treatment group were investigated by visual assessment of clustering on principal coordinates analysis (PCoA) plots, and by analysis of similarity (ANOSIM) [38] calculated on unweighted and weighted UniFrac distance metrics [39] using QIIME scripts. When significant differences were observed (P < 0.05), PRIMER (v 7.0) was used to conduct pairwise ANOSIM to determine which time-points were significantly different.

The count table of OTUs was then analyzed to determine differentially expressed OTUs over time. For these purposes, sequencing data were prepared as described above, but then sequences were clustered into OTUs using a closed-reference OTU picking protocol at the 97% sequencing identity level in order to determine which known types of bacteria were altered with treatment. Differentially expressed genes were determined using EdgeR based on the matrix of OTU counts and were not rarefied to an even sampling depth but instead normalized using EdgeR function *calcnormfactors* [40, 41]. An OTU was considered differentially expressed if the false discovery rate (FDR) value of P was < 0.05.

The software Phylogenetic Investigation of Communities by Reconstruction of Unobserved States (PICRUSt) was used to predict the metagenome [42]. The resulting OTU table was normalized by the expected copy number(s) of the 16S rRNA gene in each OTU. PICRUSt was then used to predict the metagenome. Each sample was rarefied to an even sequencing depth to adjust for uneven sequencing depth prior to further analysis. Differences in the metagenomes among the groups were investigated by visual assessment of clustering on non-metric multi dimension scaling (NMMDS) plots and by analysis of similarity (ANOSIM) calculated on Bray Curtis dissimilarity performed in PRIMER [43]. Differentially expressed Kyoto Encyclopedia of Genes and Genomes (KEGG) orthologies were determined using the predicted metagenome (*i*.*e*., counts of KEGG orthologies) within EdgeR as described above with a KEGG orthology (KO) being considered differentially expressed if the FDR value of P was < 0.05. These were then mapped using KEGG database to determine which inferred metagenomics pathways were altered within each treatment group over time [44–46].

## Results

### Open reference OTU picking and analysis

Given the likelihood that many of the microbial 16S rRNA reads found in equine stool are unknown, alterations in the microbiota were evaluated using an open reference OTU platform to examine as many of the reads as possible. Initially, low frequency OTUs (*i*.*e*., OTUs represented < 10 times) or OTUs present in < 9 animals were removed. To account for differences in sequencing depth, an even sequencing depth of 16,000 reads per sample was selected. Alpha rarefaction curves had plateaued at this sampling depth and Good’s coverage index estimates indicated that over 90% (median, 93.5%) of the species were represented at this sampling depth, with no differences among treatment groups or time (S1A and S1B Fig). Following quality filtering, OTU picking, removal of ultra-low abundance OTUs, and selecting an even sampling depth of 16,000 reads/sample, all samples remained for analysis. Each treatment group was analyzed independently in order to assess how the fecal microbiota changed over time in response to treatment (*i*.*e*., placebo, phenylbutazone, or firocoxib).

### Alpha diversity

**Fig 1:**
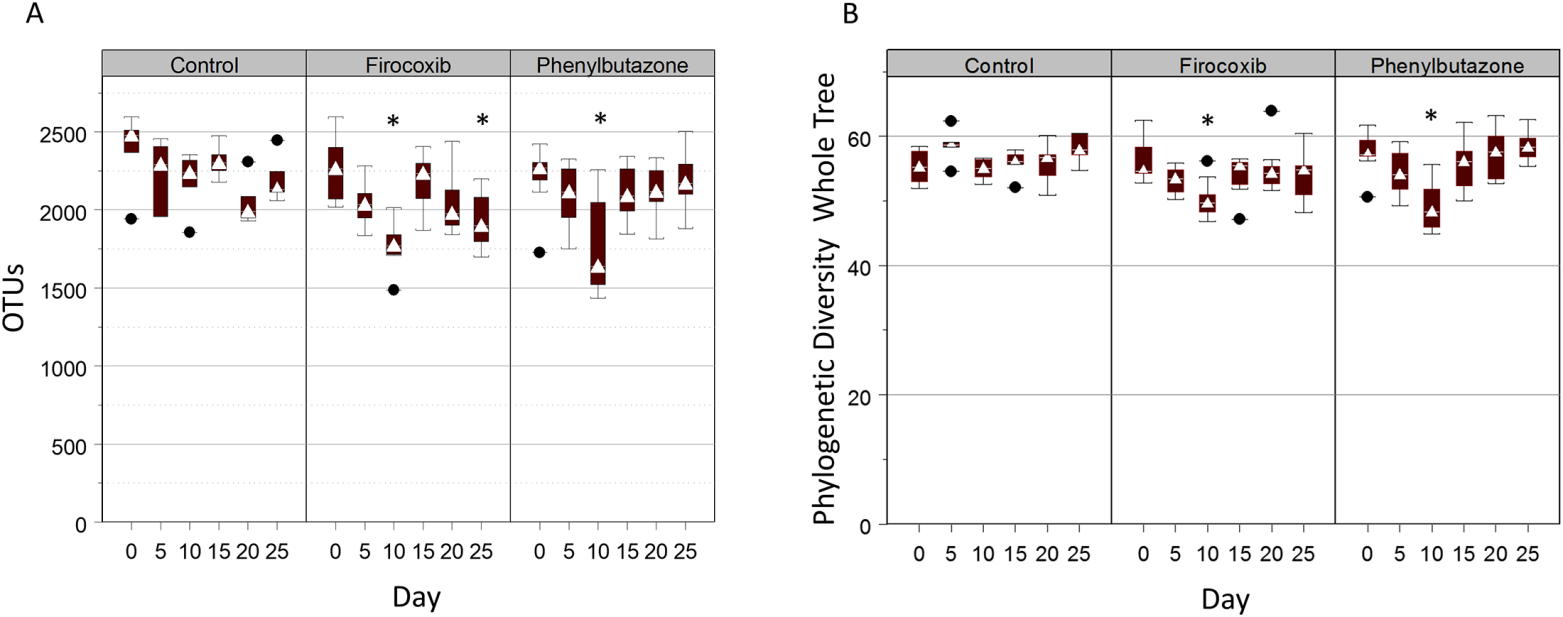
NSAID administration results in a temporary decrease of richness and alpha diversity of the fecal microbiota. A) Numbers of OTUs by day of treatment. White triangle = median; bottoms and tops of boxes are 25^th^ and 75^th^ percentiles, respectively. Whiskers extend to a fixed multiple of the interquartile distance, and black circles with horizontal lines are outliers. B) PD whole tree diversity index by day of treatment. White triangle = median; bottoms and tops of boxes are 25^th^ and 75^th^ percentiles, respectively. Whiskers extend to a fixed multiple of the interquartile distance, and black circles with horizontal lines are outliers. Data were analyzed with generalized linear models with the dependent (outcome) variables of number of OTUs or phylogenetic diversity (PD) whole tree index; independent variables of time, group, and their interaction were modeled as fixed categorical variables (ordered in the case of time) and individual horse was modeled as a random effect. Post hoc multiple comparisons among groups and times were adjusted using the method of Sidak. Asterisks above the bar represent significant differences as compared with day 0.

There were significant effects of time that varied by group. Considering effects of group within time, horses treated with firocoxib and phenylbutazone had significantly (P < 0.05) lower numbers of OTUs than control horses on day 10, and firocoxib horses had significantly lower observed species on day 25 than horses treated with phenylbutazone; no other differences were detected between groups at any other times (Fig 1A). Considering the effects of time within group, there were no significant differences among times for control horses. For horses in the firocoxib group, numbers of OTUs were significantly (P < 0.05) lower on days 10 and 25 than day 0, and values on day 10 were significantly (P < 0.05) lower than those on day 15. For horses in the phenylbutazone group, values on day 10 were significantly (P < 0.05) lower compared to all other time points, but no other differences between pairs of times were significant.

We then examined the PD whole tree diversity index, which accounts for both richness and phylogenetic distance of observed OTUs [47, 48]. There were significant effects on the PD whole tree diversity index of time that varied by group. Considering the effects of group within time, horses treated with firocoxib and phenylbutazone had significantly (P < 0.05) lower PD whole tree diversity indices than controls on day 5 and 10; no other significant differences were detected between groups at any other times (Fig 1B). Considering effects of time within group, there were no significant differences among times for control horses. For horses in the firocoxib group, PD whole tree values were significantly (P < 0.05) lower on day 10 than on either day 0 or day 20; no other differences were significant. For horses in the phenylbutazone group, PD whole tree values on day 10 were significantly (P < 0.05) lower than those on all other days, but no other differences between pairs of times were significant. Taken together, these data indicate that NSAID administration temporarily reduced the alpha diversity of the fecal microbiota of adult horses irrespective of COX selectivity. The effects of phenylbutazone were most notable at day 10, whereas the effects of firocoxib were significant at multiple days but the magnitude of effect was greatest at day 10.

### Beta diversity

**Fig 2:**
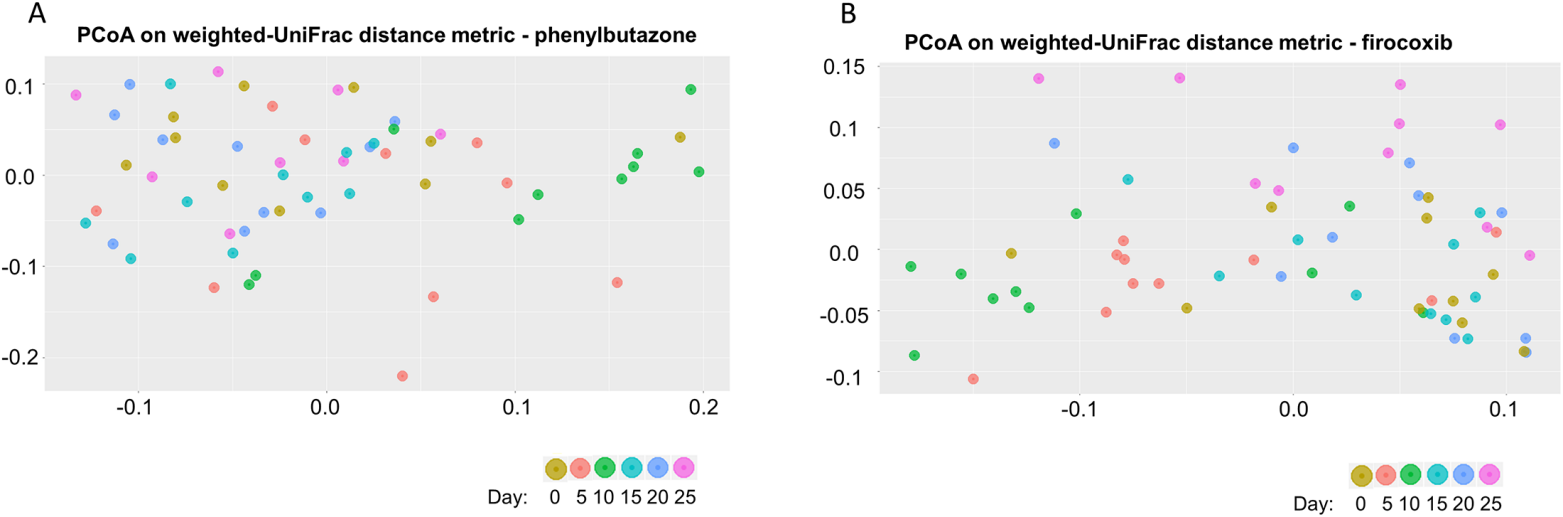
NSAID administration results in a transient alteration of the fecal microbiota as determined by beta diversity measures. A) PCoA plot based on the weighted UniFrac distance measure of the fecal microbiota of phenylbutazone-treated horses colored by each sampling time-point. ANOSIM based on this distance metric revealed significant differences among the time-points within the phenylbutazone group, with these differences attributed to day 10 compared to all other days. B) PCoA plot based on the weighted Unifrac distance measure for each sample within the Firocoxib group. Each time-point is colored based on the legend to the right. ANOSIM based on this distance metric revealed significant differences among the time-points within the firocoxib group, with these differences attributed to day 10 compared to all other days.

Principal coordinate analysis (PCoA) plots based on the weighted UniFrac distance metric revealed no apparent visual clustering of control animals at any time (S2 Fig). This was substantiated quantitatively by analysis of similarity (ANOSIM) based on the UniFrac distance metric which revealed no significant difference among the time-points within the control group (R value = 0.008; P value = 0.387). PCoA plots based on the weighted UniFrac distance metric revealed an apparent visual clustering of day 10 samples within the phenylbutazone group (Fig 2). Indeed, ANOSIM based on the UniFrac distance metric revealed significant differences among the time-points within the phenylbutazone group (R value = 0.185; P value = 0.001). Pairwise ANOSIM revealed that a significant difference among groups in the UniFrac distance metric wasobserved only for day 10 (Table 1). Similarly, PCoA plots based on the weighted UniFrac distance metric which revealed an apparent visual clustering of sample days within the firocoxib group (Fig 2). ANOSIM based on the UniFrac distance metric revealed significant differences among the time-points within the firocoxib group (R value = 0.17; P value = 0.001). Pairwise ANOSIM revealed this difference was primarily related to differences in day 10 relative to all other time-points and, to a lesser extent, day 5 versus all other days (Table 2). Although there were significant (P <0.05) differences among other days, the R values were quite small (< 0.3) indicating minimal magnitude of these differences. Interestingly, the greatest difference existed between day 10 and day 25 in this group perhaps suggesting continued adaptation of the microbiota after cessation of NSAIDs.

**Table 1:** Pairwise ANOSIM R values based on weighted UniFrac distance metric from phenylbutazone-treated animals.

Times	P0	P5	P10	P15	P20	P25
P0						
P5	0.092					
P10	**0.252**	0.113				
P15	0.028	0.113	**0.438**			
P20	0.033	**0.17**	**0.479**	-0.056		
P25	0.053	0.129	**0.355**	0.1	0.053	

Pairwise ANOSIM R values from pairwise comparison of each time-point within the phenylbutazone treatment group. R values in bold were significantly different than 0 (P < 0.05).

**Table 2:** Pairwise ANOSIM R values based on weighted UniFrac distance metric from firocoxib-treated animals.

Times	F0	F5	F10	F15	F20	F25
F0						
F5	**0.128**					
F10	**0.376**	0.115				
F15	-0.03	**0.21**	**0.42**			
F20	-0.056	**0.211**	**0.408**	-0.047		
F25	0.06	**0.271**	**0.421**	0.106	-0.009	

Pairwise ANOSIM R values from pairwise comparison of each time-point within the firocoxib treatment group. R values in bold were significantly different than 0 (P < 0.05).

### Differentially expressed OTUs

**Fig 3:**
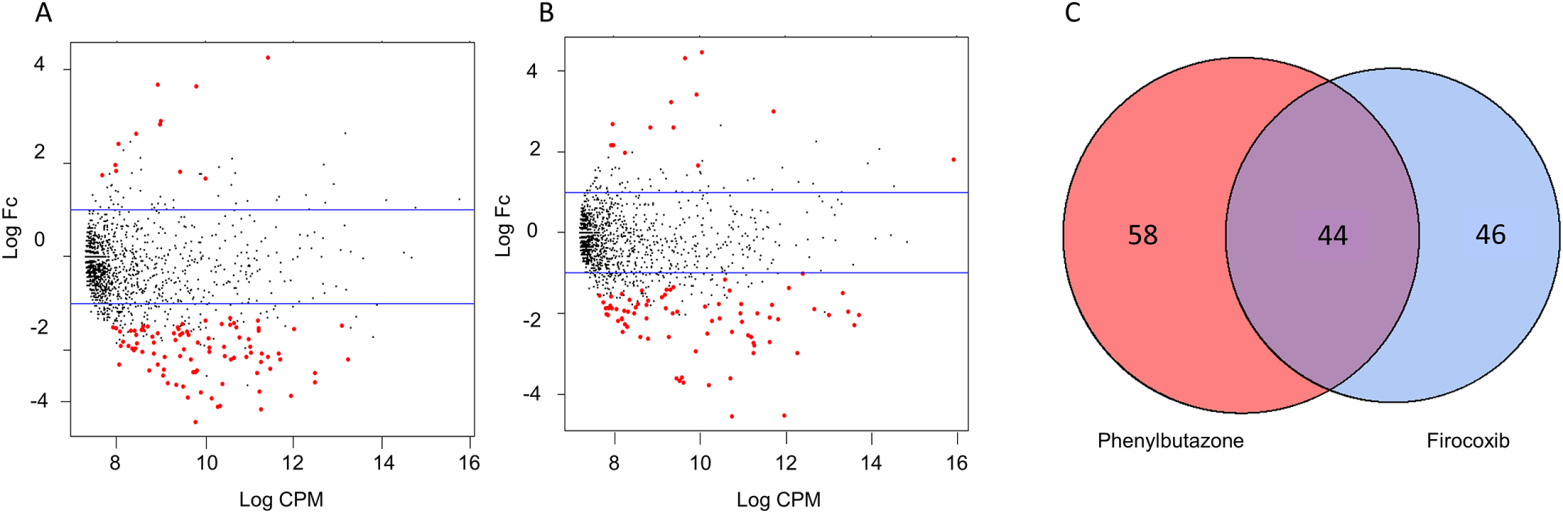
Both COX selective and non-selective NSAIDs result in similar effect size and overlap of DE OTUs. A) MA plot of OTUs in phenylbutazone-treated horses with DE OTU between days 0 and 10 colored red, with OTUs lost negative and OTUs gained positive. Blue line represents log_2_ fold-change of 1. B) MA plot of OTUs in firocoxib-treated horses with DE OTU between days 0 and 10 colored red. C) Venn diagram demonstrating overlap of DE OTUs between phenylutazone- and firocoxib-treated horses when comparing day 0 to day 10.

**Fig 4:**
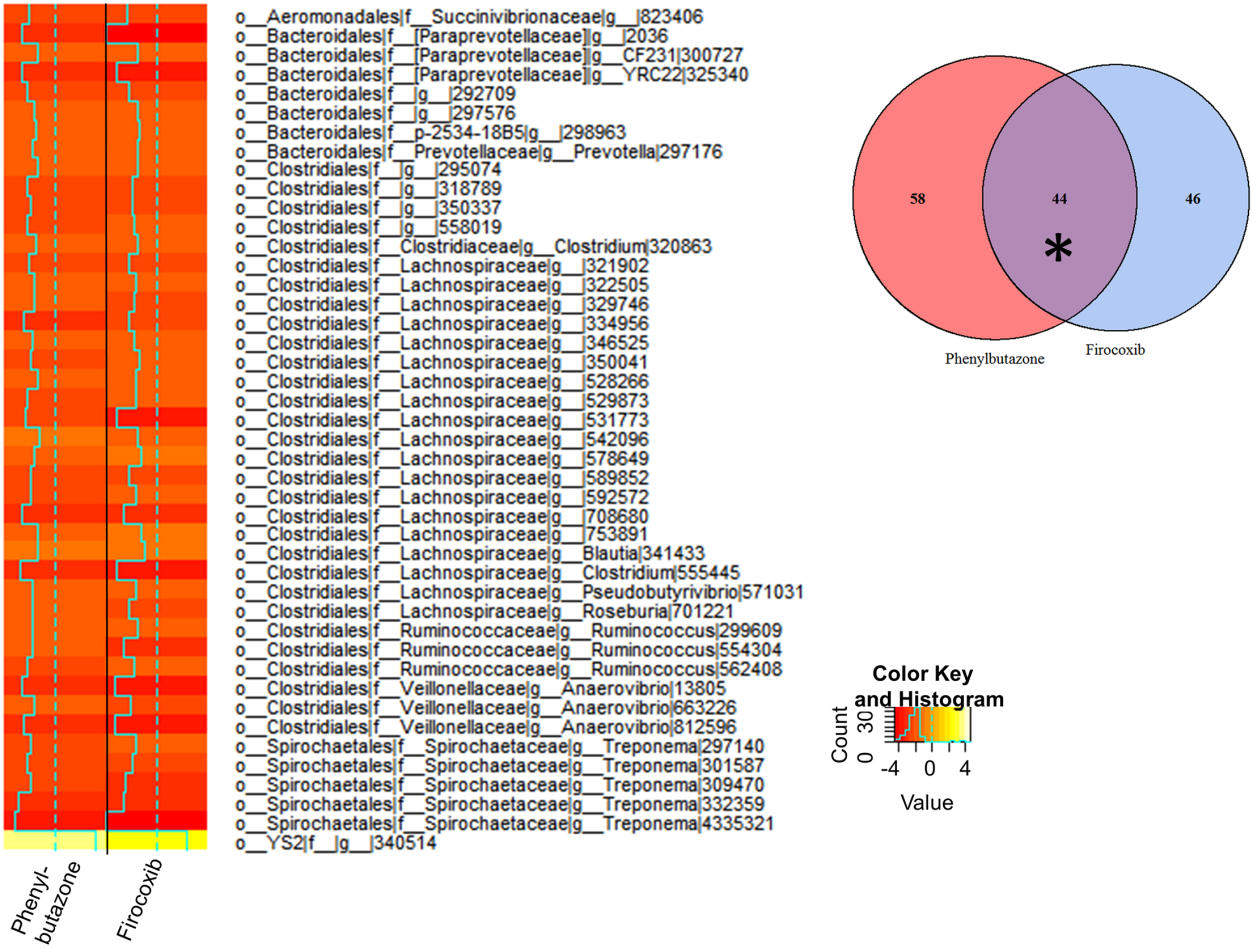
NSAID administration resulted in similar fecal microbiota changes irrespective of COX selectivity. This change was characterized primarily by a reduction in the members of the phylum *Firmicutes*, mainly *Lachnospiracea* after 10 days of NSAID administration. Heatmap showing log_2_ fold change of concordant DE OTUs between both phenylbutazone (left column) and firocoxib (right column) treated horses between days 0 and 10. Dashed line in center of each heatmap column represents log_2_ fold change of 0 (*i*.*e*., no fold change), solid line represents mean log_2_ fold change specific to each OTU within each treatment group. Asterisk in Venn diagram (inset) visually depicts the source of the 44 OTUs represented in this heatmap.

**Fig 5:**
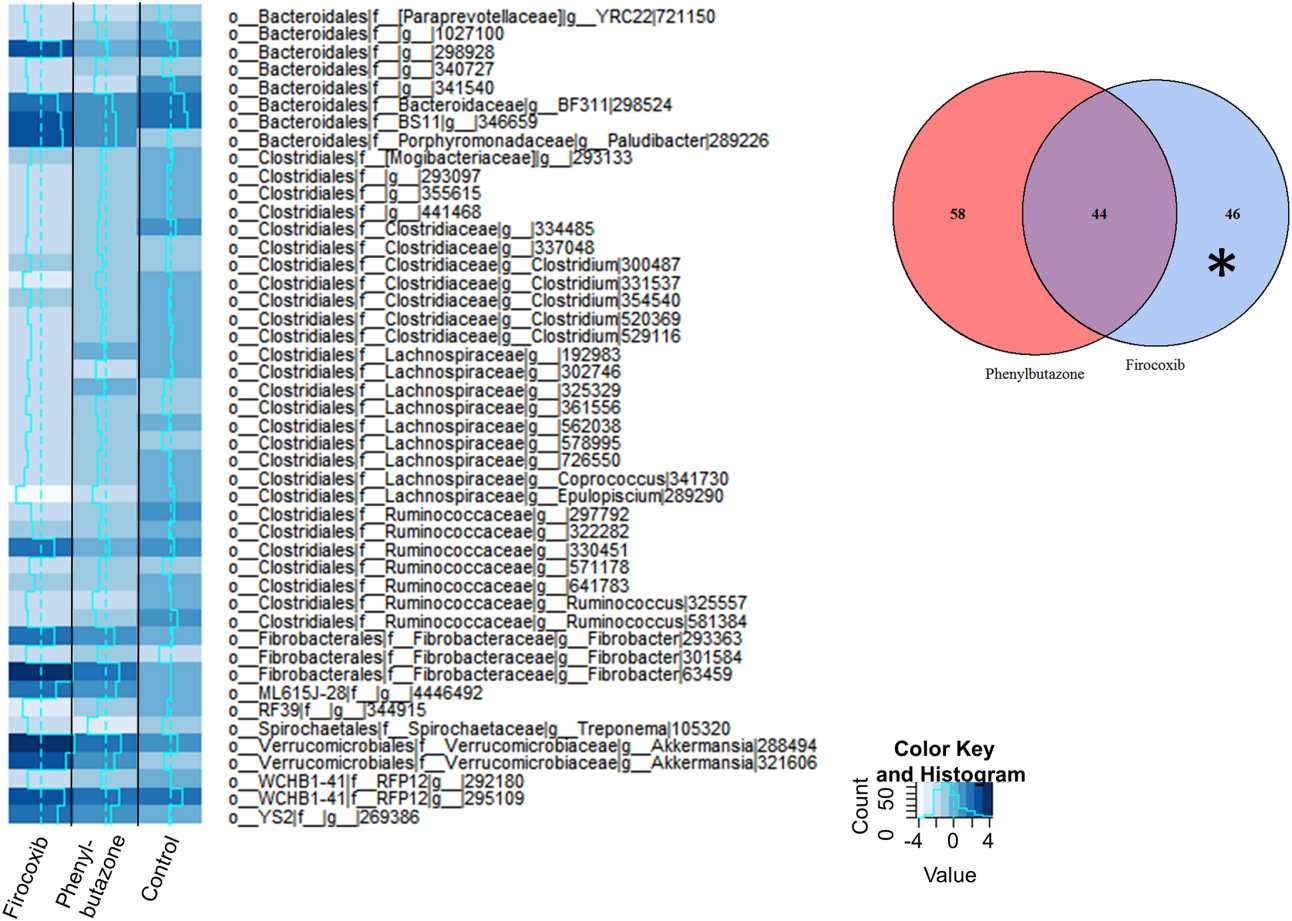
The directionality of the DE OTUs unique to the firocoxib group is mirrored in the phenylbutazone group and these OTUs represent similar families of bacteria albeit with different OTU assignment. Heatmap showing log_2_ fold-change of DE OTUs in firocoxib-treated horses only (left column) after 10 days of administration. The log_2_ fol change of these same OTUs are shown for phenylbutazone-treated (middle column) and control (right column) horses for comparison although these OTUs were not DE in these groups. Dashed line in center of each heatmap column represents log_2_ fold change of 0 (*i*.*e*., no fold change), solid line represents mean log_2_ fold change specific to each OTU within each treatment group. Asterisk in Venn diagram (inset) visually depicts the source of the 46 OTUs represented in this heatmap.

**Fig 6:**
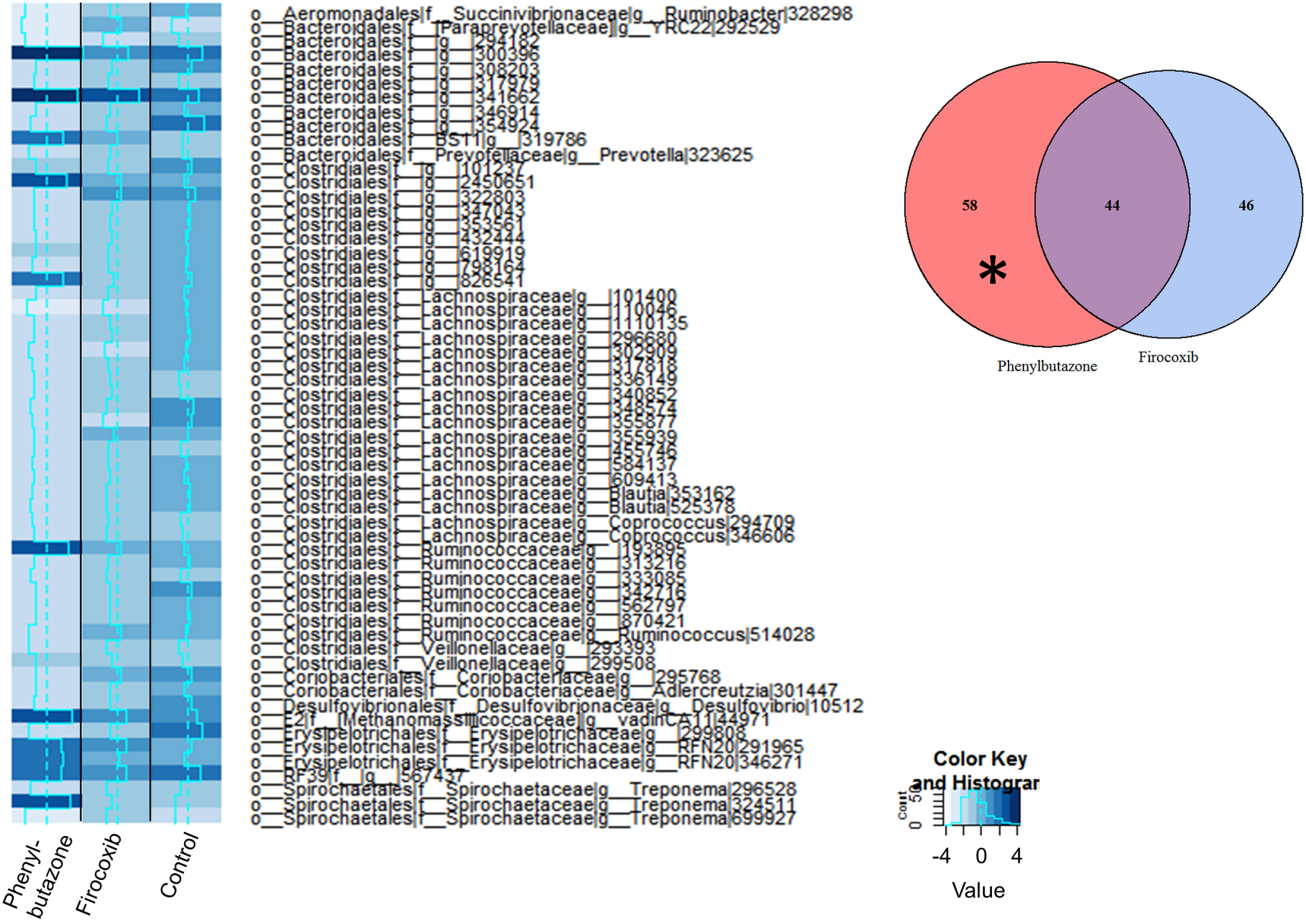
The directionality of the DE OTUs unique to the phenylbutazone group are mirrored in the firocoxib group and represent similar families of bacteria albeit with different OTU assignment. Heatmap showing log_2_ fold-change of DE OTUs in phenylbutazone-treated horses only (left column) after 10 days of administration. The log_2_ fold change of these same OTUs are shown for firocoxib-treated horses (middle column) and control horses (right column) for comparison although these OTUs were not DE in these groups. Dashed line in center of each heatmap column represents log_2_ fold change of 0 (*i*.*e*., no fold change), solid line represents mean log_2_ fold change specific to each OTU within each treatment group. Asterisk in Venn diagram (inset) visually depicts the source of the 58 OTUs represented in this heatmap.

We then analyzed these data to identify differentially expressed OTUs between day 10 and all other days. To derive meaning from these data, we utilized a closed reference-picking platform to determine which known bacteria were altered by treatment. In addition, since our analysis of the open reference platform revealed that the majority of differences were observed between days 0 and 10, we focused our analyses on that comparison. Count tables were constructed from BIOM files, normalized using the edgeR function *calcnormfactors* with the method RLE, and then analyzed for differentially expressed (DE) OTUs between days 0 and 10. An OTU was considered differentially expressed if the false discovery rate (FDR) P value was < 0.05. MA plots revealed that many of the DE OTUs were highly expressed, with similar expression values and fold-changes in both groups of NSAID-treated horses (Fig 3A and 3B). Specifically, these analyses revealed 102 DE OTUs in the phenylbutazone group (S1 Table), 90 DE OTUs in the firocoxib-treated group (S2 Table), and 1 DE OTU in control animals. There was substantial overlap of DE OTUs amongst the NSAID-treated horses (~50%) (Figs 3C and 4). This overlap in DE OTUs, however, underestimated the similarity in expression profile because the 46 OTUs that were DE only in firocoxib-treated animals (Fig 5) generally followed the same pattern of expression as those in phenylbutazone-treated animals. Similarly, the pattern of expression of the 58 OTUs uniquely DE in phenylbutazone-treated animals were generally the same for firocoxib-treated animals (Fig 6). Heatmaps of log_2_ fold-changes of DE OTUs revealed a reduction of members of the *Firmicutes* phylum, specifically the family *Lachnospiraceae* and, to a lesser extent, concomitant expansion of *Bacteroidetes* phylum, specifically *Bacteroidales*. This pattern was observed in both concordant DE OTUs between the 2 NSAID groups (Fig 4) and when examining DE OTUs unique to each treatment group (Figs 5 and 6), but not in control animals. Moreover, while exact OTU assignment was different, taxonomic classification of these reads represent very similar bacterial families (*i*.*e*., primarily *Lachnospiracea* and to a lesser extent *Clostridiacea* and *Ruminococcacaea*). Taken together, these findings suggest that administration of phenylbutazone and firocoxib at the tested dosages result in alterations of the fecal microbiota, with the most notable alterations occurring at day 10 compared to day 0.

### NSAID-induced alterations of the inferred metagenome

**Fig 7:**
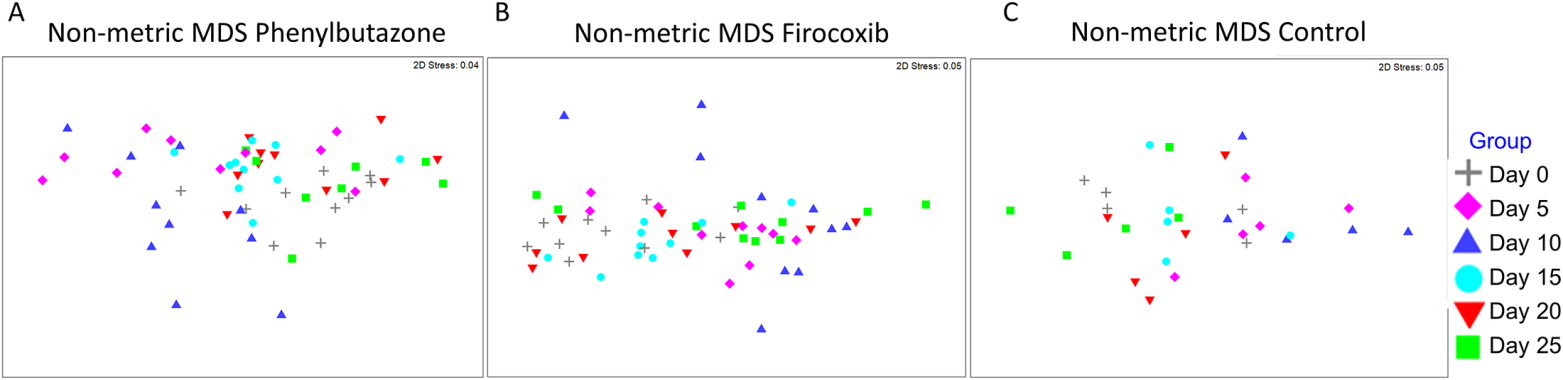
Administration of NSAIDs for 10 days, irrespective of COX selectivity, transiently alters the inferred metagenome of the fecal microbiota of adult horses. A) Non-metric MDS plots based on the Bray-Curtis dissimilarity metric of the inferred metagenome of the fecal microbiota of horses receiving phenylbutazone, colored by sampling time. B) Non-metric MDS plots based on the Bray-Curtis dissimilarity metric of the inferred metagenome of the fecal microbiota of horses receiving firocoxib, colored by sampling time. C) Non-metric MDS plots based on the Bray-Curtis dissimilarity metric of the inferred metagenome of the fecal microbiota of control horses, colored by sampling time.

In order to determine if these microbiota shifts also resulted in functional alterations of the microbiome, we used the results of closed reference OTU picking and PICRUSt to determine if NSAID-induced microbiota shifts altered the inferred metagenome. Specifically, we aimed to determine:1) if NSAIDs altered the inferred metagenome; 2) whether there were different results between COX-selective and non-selective NSAIDs; and, 3) which functional pathways were altered. ANOSIM based on the Bray-Curtis dissimilarity metric of the inferred metagenome revealed no significant differences among the control horses over time (R = 0.026; P = 0.289). A significant difference among the phenylbutazone-treated (R = 0.100; P = 0.008) and firocoxib-treated horses was observed at the different time-points (R = 0.152, P = 0.001). Specifically, non-metric MDS plots based on the Bray-Curtis dissimilarity metric revealed visual separation and clustering of day 10 samples from all other days based on inferred metagenome at the third level of the KEGG orthologies in both phenylbutazone- and firocoxib-treated animals but not control horses (Fig 7). This was confirmed quantitatively where pairwise ANOSIM based on this metric revealed that day 10 samples were substantially different from all other days with an R value > 0.3 and P < 0.05 except for day 5 in the phenylbutazone group (Table 3) and days 5 and 25 in the firocoxib group (Table 4).

**Table 3:** Table of R values resulting from pairwise ANOSIM of inferred metagenome among phenylbutazone time-points.

Times	P0	P5	P10	P15	P20	P25
P0						
P5	**0.253**					
P10	**0.477**	0.124				
P15	**0.292**	**0.126**	**0.32**			
P20	0.091	**0.13**	**0.427**	-0.013		
P25	-0.007	**0.223**	**0.545**	**0.218**	-0.001	

R values resulting from pairwise ANOSIM of inferred metagenome among phenylbutazone time-points showing that the greatest difference existed between day 10 and all other time-points (except day 5). Bolded R-values were significantly > 0 (P < 0.05).

**Table 4:** R-values resulting from pairwise ANOSIM of inferred metagenome among firocoxib time-points.

Times	F0	F5	F10	F15	F20	F25
F0						
F5	**0.278**					
F10	**0.572**	0.103				
F15	0.082	**0.224**	**0.472**			
F20	-0.02	0.064	**0.244**	0.019		
F25	**0.279**	0.012	0.096	**0.288**	0.046	

R-values resulting from pairwise ANOSIM of inferred metagenome among firocoxib time-points showing that the greatest difference tended to exist between day 10 and all other time-points although there were some notable differences between other time-points within the firocoxib group. Bolded R-values were significant (P < 0.05)

#### Differentially expressed KEGG orthologies

**Fig 8:**
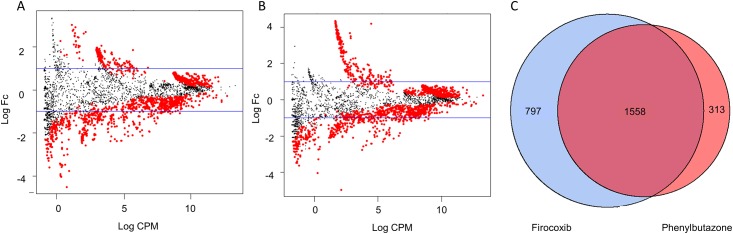
Both COX-selective and non-selective NSAIDs result in a similar effect-size and overlap of DE KOs. A) MA plot of KOs in phenylbutazone-treated horses with DE KOs between days 0 and 10 colored red, with KOs lost negative and KOs gained positive. Blue line represents log_2_ fold-change of 1. B) MA plot of KOs in firocoxib- treated horses with DE KO between days 0 and 10 colored red. C) Venn diagram demonstrating overlap of DE KOs between phenylutazone- and firocoxib-treated horses when comparing day 0 to day 10.

After determining that the greatest alteration of the inferred metagenome occurred at day 10 compared to all other days, we specifically analyzed day 10 versus day 0 inferred metagenome to determine which KEGG orthologies (**KOs**) were altered by NSAID administration. As above, count tables were constructed from BIOM files that were generated by PICRUSt of the inferred metagenome. These were normalized using edgeR function *calcnormfactors* with the method RLE, and then analyzed for differentially expressed KOs between days 0 and 10. MA plots revealed that many of the DE KOs were highly expressed with similar expression values and fold-changes in both groups of NSAID treated horses (Fig 8A and 8B). Specifically, these analyses revealed 1,871 DE KOs in the phenylbutazone group and 2,355 DE KOs in the firocoxib-treated group. There was substantial overlap of DE KOs in the NSAID-treated horses: ~81% of the DE KOs in the phenylbutazone comparison were also DE in the firocoxib comparison (Fig 8C), suggesting similar NSAID-induced alterations of the inferred metagenome. In order to determine the pathways to which these KOs belonged, the top 500 DE KOs (*i*.*e*., 500 KOs with smallest FDR P values) were uploaded to KEGG pathway analysis. Of these 500 KOs, nearly 20% affected metabolic pathways (Table 5). It appeared that there were NSAID-induced alterations in many aspects of the metabolic pathways of the fecal microbiota of these horses after 10 days of NSAID administration, with substantial overlap between firocoxib- and phenylbutazone-treated horses.

**Table 5:** Both firocoxib and phenylbutazone alter similar functional pathways.

Functional Pathways From Top 500 DE KO		
Pathway	Number of KOs that map to this pathway	
	Phenylbutazone	Firocoxib
Metabolic pathways	81	108
Biosynthesis of secondary metabolites	22	36
Microbial metabolism in diverse environments	36	42
Biosynthesis of antibiotics	14	23
Carbon metabolism	12	17
Pyruvate metabolism	10	8
Starch and sucrose metabolism	9	9
Biosynthesis of amino acids	8	7
Degradation of aromatic compounds	3	0
Glycolysis / Gluconeogenesis	5	0

Top 10 pathways of the top 500 DE KO (500 lowest FDR P values) demonstrating that NSAIDs primarily induce alteration of metabolic pathways.

## Discussion

A number of reports indicate that the microbiota is a critical component of NSAID enteropathy and that NSAID use may result in dysbiosis [49–51]. Differential effects of COX-selective and non-selective NSAIDs on NSAID-induced dysbiosis has not been examined previously, nor have the effects of NSAIDs in the microbiota of horses. Here, we show for the first time that clinically-relevant duration and dosages of NSAIDs result in a transient dysbiosis of the fecal microbiota of healthy, adult horses. Importantly, we have demonstrated similar effects of non-selective and COX-2-selective NSAIDs on the equine microbiota. These changes were primarily characterized by loss of members of the *Firmicutes* phylum, specifically the family *Lachnospiraceae* and, to a lesser extent, the families *Clostridiacea* and *Ruminococcacaea*. NSAIDs are among the most frequently administered classes of medications in equine health care. Given the frequency of use of this class of medication, understanding their impact on the equine microbiota is clinically important because alteration of the equine GI microbiota has been linked to inflammatory diseases including colitis and laminitis [9–12].

Deleterious effects of NSAIDs on the equine GI tract are well-recognized. The current paradigm of the pathophysiology of NSAID-induced GI damage proposes that COX-2-selective NSAIDs should be safer for the equine GI tract. In human medicine, COX-2-selective NSAIDs have successfully reduced the incidence of NSAID-induced gastric ulcers. However, the incidence of NSAID-induced lower GI damage remains unchanged despite increased use of COX-2-selective NSAIDs, suggesting that this class may not necessarily provide a safer alternative for NSAID enteropathy in people [23]. It remains unclear why COX-2 selective NSAIDs do not reduce the incidence of NSAID enteropathy in people. Some studies have suggested that both COX-1 and COX-2 are constitutively expressed in various tissues and that both isoforms contribute to the maintenance of GI homeostasis this inhibition of either isoform has deleterious effects [52]. Our findings suggest that, in horses, both selective and non-selective COX inhibitors result in a similar dysbiosis although the clinical significance of this dysbiosis remains unclear. Conflicting results have been obtained regarding the role of NSAID-induced dysbiosis. Xiao *et al*. demonstrated that NSAID-induced dysbiosis protects mice against NSAID enteropathy, but others have demonstrated opposite results [53–58].

The mechanism by which NSAIDs cause dysbiosis is unknown. One possibility is that reactive oxygen species (ROS) released from intestinal epithelial cells following NSAID-induced mitochondrial damage contributes to this dysbiosis, as ROS molecules released during intestinal inflammation have been shown to induce a dysbiosis [59]. In rodent models and *in vitro* studies, NSAIDs have been shown to induce a topical effect on the intestinal mucosa whereby NSAIDs enter the enterocyte and affect numerous organelles including mitochondria and the endoplasmic reticulum eventually leading to ROS generation [60–63]. When ROS molecules react with luminal substrates such as thiosulphates, they can subsequently act as electron receptors for pathogens that use respiration, which allows the respiring bacteria to outcompete commensal bacteria that use fermentation for energy generation [64]. This results in loss of obligate anaerobes with expansion of facultative anaerobes and aerobes. The dysbiosis we observed was characterized by loss of *Lachnospiracea*, and to a lesser extent *Clostridiacea* and *Ruminococcacaea*, all obligate anaerobes. These findings suggest that NSAID-induced dysbiosis might cause inflammation resulting in increased ROS production. While not linked to dysbiosis, increased ROS production has been demonstrated during NSAID-induced intestinal injury [64, 65]. Another mechanism by which NSAIDs might induce a dysbiosis is the antimicrobial properties of some NSAIDs. It is unknown, however, whether phenylbutazone or firocoxib exhibit antimicrobial properties, especially because the GI luminal concentrations of these drugs in horses (or other species) remains unknown [66–68].

The clinical significance of the NSAID-induced dysbiosis remains elusive. While the horses enrolled in this study exhibited no signs of illness, based on daily physical examination, there was no attempt made to document injury to the GI tract. The types of bacteria altered by NSAIDs in this study have been linked to disease in horses. Specifically, members of the family *Lachnospiraceae* were higher in healthy horses than in horses with colitis [69]. In addition, *Clostridiales* families *Clostridiaceae* and *Lachnospiraceae*, both of which were reduced following NSAID administration, have been designated as integral players in the maintenance of mucosal homeostasis, particularly due to their ability to produce large quantities of the short chain fatty acid butyrate [18, 70]. It is unknown if NSAID administration resulted in decreased butyrate production or alterations in the gut metabolome of these horses. Nevertheless, our data suggests that dysbiosis may significantly impact the function of the microbiota, because we demonstrated functional differences in the inferred metagenome after 10 days of NSAID administration with similar changes between the 2 classes of NSAIDs. Limitations of the taxonomic resolution of 16S sequencing prevent further classification of the bacteria altered by NSAIDs beyond family and some genera assignment. Our results, however, demonstrate a great deal of concordance of the effects of NSAIDs on the fecal microbiota of adult horses. These findings suggest that NSAID administration over 10 days temporarily alters both the population and the function of the microbiota. The implications of these changes on host health remain unknown.

This study has several limitations. First, the sample size was relatively modest. Nevertheless, these data represent the largest and only study examining the effects of NSAID administration on the fecal microbiota of horses, and contribute important findings, irrespective of species, on the impact of COX-2-selective versus non-selective NSAIDs on the fecal microbiota. Second, we examined only the fecal microbiota. Although fecal samples are often used as indicators of the intestinal microbiota, it has been shown that fecal microbiota data do not always correlate with samples taken from regions of the tract, and that GI variation in the microbiota occurs among different anatomical sites of the intestinal tract [71]. For example, fecal samples might not have reflected the microbiota inhabiting the intestinal mucosa; the mucosal microbiota might be more important for immune development and modulating host inflammatory responses because it is more intimately associated with the immune cells of the host’s intestinal tract. Third, our population was limited to a single equine facility and consisted of healthy horses. We do not know to what extent our results from these horses can be extrapolated to other populations of horses, particularly sick horses or horses being treated concurrently with other medications including antimicrobials. Finally, the metagenome of the microbiota was inferred rather than directly assayed. While the inferred metagenome has been shown to correlate with the true metagenome and is used routinely for these purposes, more robust and detailed information could be gathered by assaying the actual metagenome rather than the inferred metagenome [72, 73]. By utilizing the inferred metagenome it is difficult to assign specific function to specific bacteria to more precisely understand how the relative loss or gain of a specific bacteria might affect the metagenome. Similarly, we utilized 16S rRNA sequencing which, while well-accepted, has several well-recognized limitations that prevented deeper taxonomic resolution. Other approaches to query the microbiota exist, including whole genome sequencing or prior culture. These approaches could have allowed a more complete understanding of the community and function of the microbiota in our study.[74] A further limitation was that only a single dosing regimen of each drug was examined in this study. Each drug was dosed according to label directions, but many different dosing regimens are used clinically, including the use of a loading dose of firocoxib [75]. Future studies evaluating differences in efficacy and GI injury using different dosing protocols of these and other NSAIDs is warranted in order to determine if any approach is safer and more efficacious than another. A final limitation of this work is the relatively descriptive nature of this work. The clinical ramifications of NSAID-induced microbiota changes are unknown; however, documenting the effects of this class of medications on the equine microbiota is the first necessary step in understanding how these changes contribute to equine health and whether these changes render horses more or less susceptible to the effects of NSAIDs on the GI tract.

This work suggests that NSAIDs, ***irrespective of COX selectivity***, transiently alter the fecal microbiota and inferred metagenome of adult horses when administered for 10 days. These findings add to the increasing body of evidence suggesting that NSAIDs induce dysbiosis in both people and animals although the implications of this dysbiosis remain unclear. In addition, our findings demonstrate that NSAID-induced dysbiosis is similar for both COX selective and non-selective NSAIDs in horses. Deciphering the complex interaction of NSAIDs, the host, and the microbiota in both equine health and disease is the focus of ongoing and future work.

## Supporting information

S1 Fig 16,000 reads per sample is adequate sampling depth. **A)** Goods coverage estimates for each treatment group at each time-point. **B)** Alpha rarefaction curves for each treatment group at each time suggests that 16,000 reads per sample provides an adequate sampling depth.
(TIF)

S2 Fig The fecal microbiota of horses administered placebo changed minimally over time. PCoA plot based on the weighted UniFrac distance measure showing lack of clustering within the control group as determined by visual appearance and ANOSIM (R = 0.0078, P = 0.387).
(TIF)

S1 Table List of DE OTUs in phenylbutazone-treated horses between days 0 and 10. (XLSX)

S2 Table List of DE OTUs in firocoxib-treated horses between days 0 and 10. (XLSX)
