# Supplementary figures and images for: Differential effects of selective and non-selective cyclooxygenase inhibitors on fecal microbiota in adult horses
Source: PLoS One. 2018 Aug 23;13(8):e0202527. doi: 10.1371/journal.pone.0202527 (PMC6107168; doi:10.1371/journal.pone.0202527)

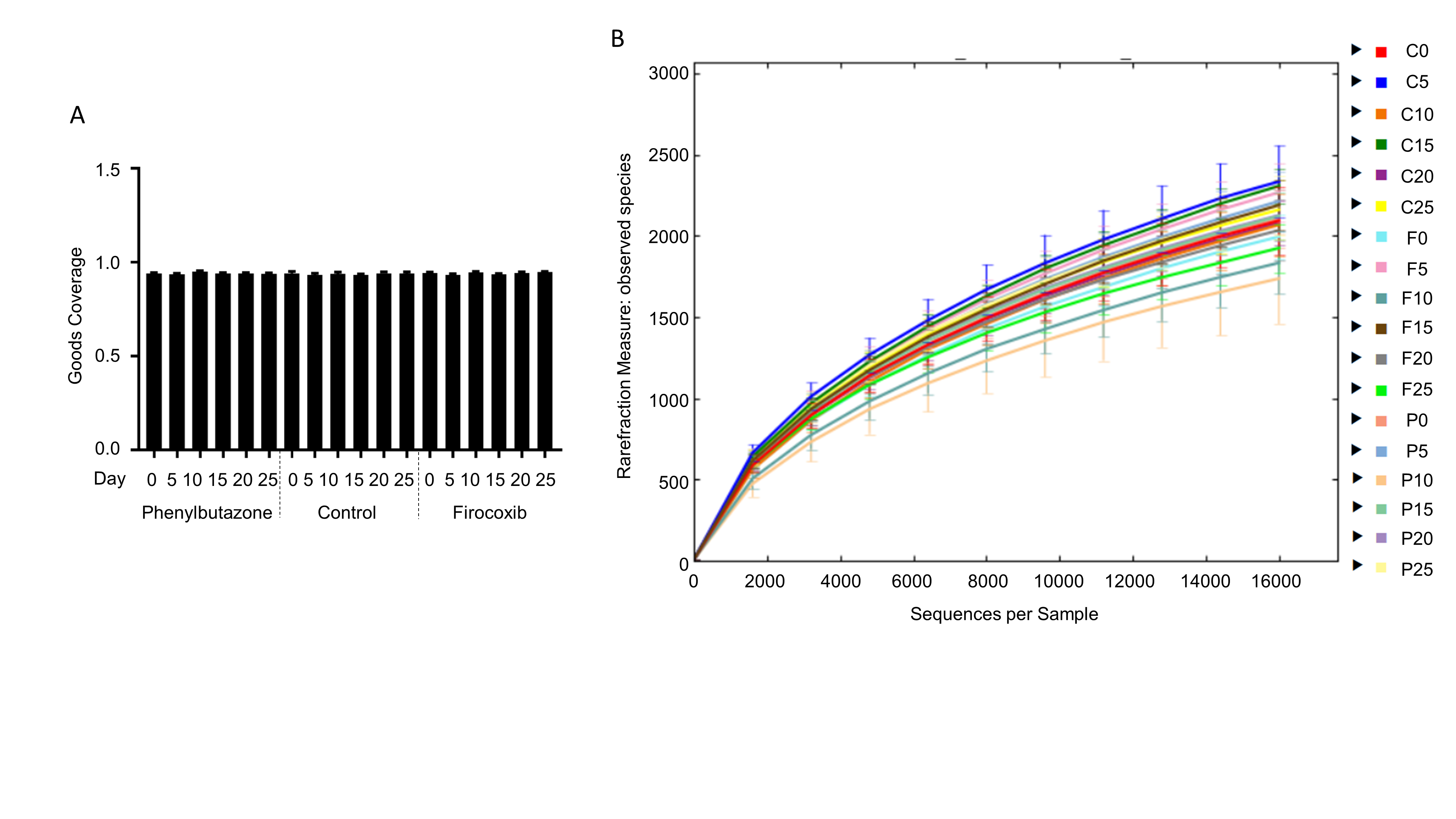

Supplement: S1 Fig — A) Goods coverage estimates for each treatment group at each time-point. B) Alpha rarefaction curves for each treatment group at each time suggests that 16,000 reads per sample provides an adequate sampling depth. (TIF) [file pone.0202527.s001.tif]

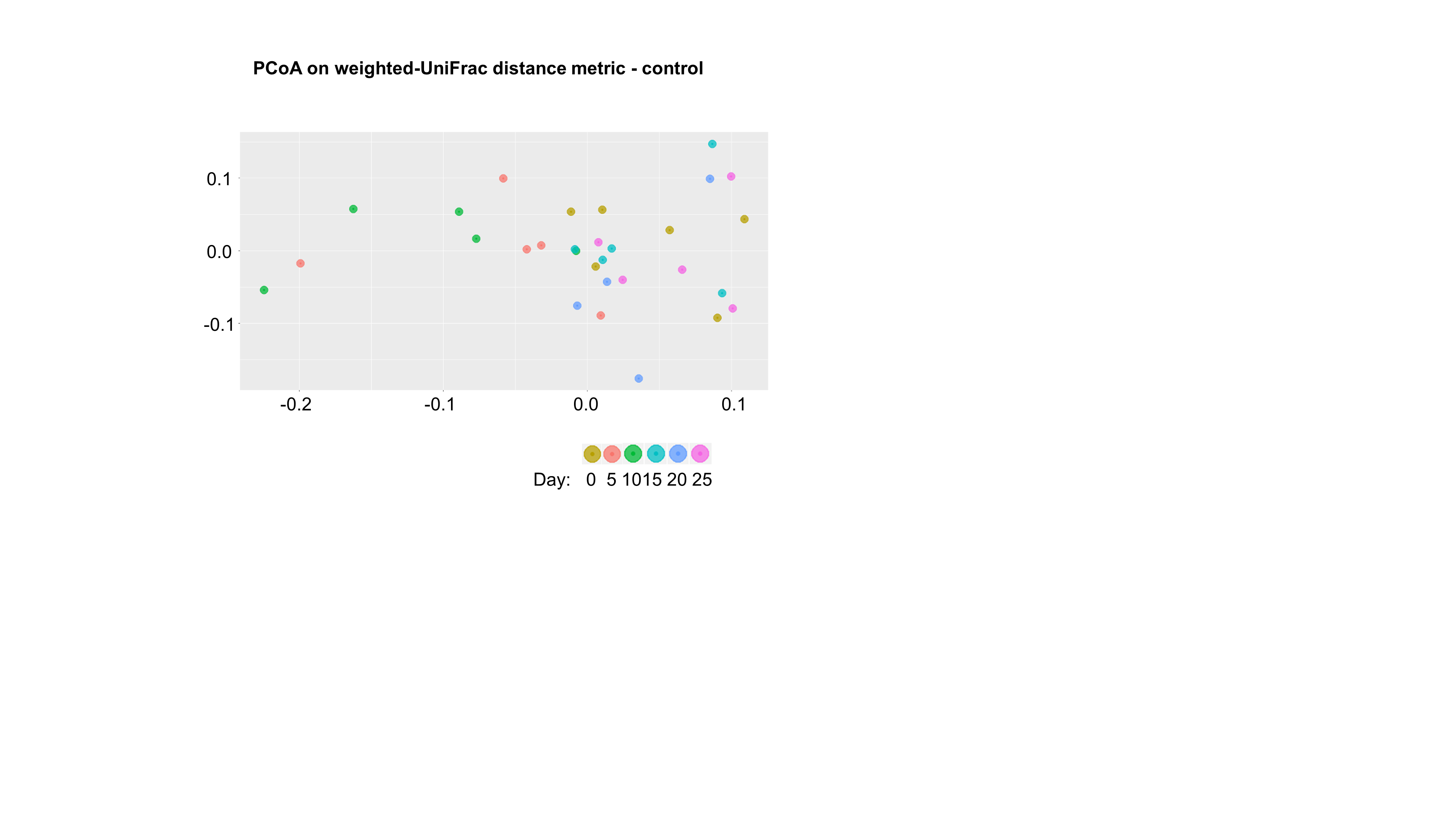

Supplement: S2 Fig — PCoA plot based on the weighted UniFrac distance measure showing lack of clustering within the control group as determined by visual appearance and ANOSIM (R = 0.0078, P = 0.387). (TIF) [file pone.0202527.s002.tif]
